# A Qualitative Study Examining the Application of Compression Therapy for Inpatients With Venous Leg Ulcers—Perspectives of Hospital Staff Where It Is Routinely Applied

**DOI:** 10.1111/iwj.70810

**Published:** 2026-02-03

**Authors:** Yaping Lian, Linda Birt, Fiona Poland, Felix Naughton, Christine Moffatt, David Wright

**Affiliations:** ^1^ University of Leicester Leicester UK; ^2^ Tissue Viability Team Cambridge University Hospitals NHS Foundation Trust Cambridge UK; ^3^ School of Healthcare, College of Life Sciences University of Leicester Leicester UK; ^4^ School of Health Sciences University of East Anglia Norwich UK; ^5^ Nottingham University Hospital Nottingham UK

**Keywords:** compression bandage, compression therapy, hospital clinicians, qualitative, venous leg ulceration

## Abstract

Compression therapy is the cornerstone, first‐line effective evidence‐based treatment for healing and managing venous leg ulcers. However, compression therapy is inconsistently applied in hospitals. This paper explores the experiences of a diverse group of clinicians and senior managers applying compression therapy in hospitals across the United Kingdom. A semi‐structured qualitative interview study was conducted with 19 participants, drawn from a larger study, who confirmed that their respective hospitals apply compression therapy to inpatients with venous leg ulcers. The interviews were analysed using reflexive thematic analysis. Analysis generated four key themes: Patients ‘slip through the net’, Prioritisation in Hospital Care, A ‘blind Spot’ within Healthcare System and Motivation to Deliver Care. Patients ‘slip through the net’ refers to inpatients with venous leg ulcers face unequal access to compression therapy both within and between hospitals. Prioritisation in Hospital Care indicates how certain diseases are given greater emphasis within healthcare systems. A ‘blind Spot’ in Healthcare System described failing to implement compression therapy has created a critical underlying ‘blind spot’ within the NHS healthcare systems. Motivation to Deliver Care refers to a deep commitment to providing compression therapy, driven by clinicians' inherent dedication and ethical obligation towards improving patient quality of care. The study identified key challenges influencing the application of compression therapy in acute hospitals despite its routine use. These include the lottery of care, systemic inequities, unclear ownership, interprofessional disputes and organisational priorities. It also demonstrated the significant role of passion, motivation and moral responsibility encouraging clinicians to implement compression therapy despite these systemic barriers.

## Implications for Clinical Practice

The study emphasised the urgent need to explore the systemic challenges inherent in inpatient venous leg ulcer care. Once this is addressed, establishing a standardised, evidence‐based care pathway with clearly defined roles and responsibilities will be paramount to ensure that all inpatients with venous leg ulcers receive timely and appropriate venous leg ulcer management in acute hospitals.

## 1 Introduction

A leg ulcer is defined as an open wound between the knee and ankle joint that remains unhealed for at least 4 weeks [1]. In the United Kingdom (UK), approximately 730 000 people developed leg ulcers in the study year 2012/2013, equating to 1.5% of the adult population [2, 3]. Venous leg ulcers (VLUs) are the most common type, accounting for over 70% of all leg ulcer cases [4]. Evidence and national guidelines consistently identify compression therapy as the cornerstone, first‐line effective evidence‐based treatment for healing and managing VLUs [1, 5, 6, 7, 8, 9]. Compression therapy is a medical treatment involving the application of pressure to the lower limbs using bandages, stockings, hosiery or wraps [6]. The therapeutic value of compression therapy for VLUs management has been recognised for centuries, evolving from rudimentary bandaging techniques to modern evidence‐based interventions for managing VLUs [10]. This treatment can reduce pain, increase healing rates, improve quality of life and enhance patients' mobility and functional status [1, 5, 6, 7, 8, 11]. Clinical evidence shows compression therapy can heal 83% of venous ulcers and prevent ulcer recurrence by 70% [12, 13, 14].

Despite robust evidence supporting its effectiveness, patients with VLUs are often not treated with compression therapy in hospital settings [15]. A 2020 literature review found that while compression therapy is widely used in community care, its use is significantly less in secondary care [16]. When patients with VLUs are admitted to hospitals, their existing compression therapies are frequently discontinued [13, 15]. A national online survey of wound care specialists revealed that only 32% (32 out of 101) reported applying compression therapy for their patients during hospital stays [15].

Inpatient management of venous leg ulcers commonly occurs in hospital medical wards where patients are admitted for acute conditions and often present with complex medical needs. In contrast, venous leg ulcer management in community settings is usually delivered in the patient's home, local health centres or residential homes by community nursing teams. Inpatient venous leg ulcer management differs from community care for several reasons. Firstly, hospitalised patients are typically admitted for acute conditions which may deprioritise chronic venous leg ulcer care. Secondly, ward nurses often work within time‐pressured, task‐driven environments. In contrast, community services provide planned visits with more time for thorough assessment, regular review and continuity of care. Community nurses often have greater expertise in leg ulcer management and follow structured pathways. As a result, compression therapy is typically more consistent and effective in community settings than on hospital wards. The discrepancy in care delivery highlighted a substantial gap between opportunity and service delivery in this area, including the delivery of sustained compression therapy to treat patients with VLUs within the inpatient environment [16].

A systematic review conducted in 2023 [17] found only four studies exploring hospital clinicians' views and experiences of using compression therapy for inpatients with VLUs. Three key themes were generated: educational needs arising from limited clinical procedure knowledge, patient factors influencing adherence and organisational resources including availability of suitable equipment and trained staff. Although these studies offer some insights, the mixed community and hospital settings restricted participants' focus on the perspectives of hospital clinicians. Hence, the aim of this paper is to explore the perspectives of a diverse group of clinicians and senior managers who are applying compression therapy in hospitals across the UK.

## 2 Materials and Methods

### 2.1 Methodological Orientation

A qualitative study involving healthcare professionals and senior managers in secondary care was conducted over a 1‐year period from May 2024 to June 2025. Qualitative methodology was chosen as an appropriate explorative research approach to investigate why compression therapy is underused in secondary care [18]. To fully comprehend the reasons behind its limited use, it is essential to engage with and explore the views and experiences of clinicians and senior managers across diverse hospitals and clinical roles. Clinicians are the care providers in hospital settings and possess the experience, knowledge and understanding of why hospital care occurs and does not. Their experiences are meaningful within the context of hospital settings, cultures and practices [19], which can be explored through listening to their talks and asking questions. Therefore, to capture these insights, the study employed qualitative semi‐structured interviews to facilitate the exploration of clinicians' views and experiences across a variety of clinical roles in different hospital environments.

To aid the study design, input was sought from Patient and Public Involvement (PPI) members who were patients living with VLUs or caring for someone with VLUs. Their input during the study's preparation phase influenced the recruitment of study participants by pointing out the need for participants to be well represented across different types of hospital, different job roles and different working experience in NHS hospitals.

### 2.2 Participant Recruitment and Sampling

Purposive sampling was used to select hospital clinicians, senior managers and other hospital staff involved in the care of inpatients with VLUs based on key characteristics: job role, years of hospital working experience and the region of the UK where practicing. Participants were recruited across the UK through professional social media channels, conference networking events and professional networks. The professional social media channels include Tissue Viability Nurses UK Facebook, Lower Limb Clinicians 2gether Facebook, Clinical Leadership for Lower Limb Self Care. The conference networking events include European Wound Management Association (EWMA) conference, Society of Tissue Viability conference, The Royal Society of Medicine Venous Forum Annual Scientific meeting, Wounds UK Annual conference, The Vascular Societies' Annual Scientific meeting and Leg Ulcer Forum. Professional networks include Society of Tissue Viability, Society of Vascular Nurses, Vascular Surgery Clinical Trials Network, British Geriatrics Society.

Recruitment method was also expanded to recruit participants by choosing hospitals in England at random. The random hospitals were selected from four groups of NHS Trusts stratified using ‘Social Deprivation Index’ and the Lower‐layer Super Output Areas (LSOAs) from the UK government websites. Study advertisements included a Participant Information Sheet (PIS), along with a link and a QR code to invite potential participants to express interest in interview participation. The expression of interest and availability survey was conducted via a Microsoft Forms survey. The clinicians’ group includes a mix of doctors, nurses and senior managers from various clinical roles and genders. Participants were excluded if they were not working in hospital settings. Recruitment took place for a larger study and the participants for this paper are those who confirmed that their respective hospitals applied compression therapy for inpatients with VLUs.

### 2.3 Data Collection

The semi‐structured interviews took place online using Microsoft Teams meeting. Only the researcher (YL) and the participants were present at the interviews. The interview topic guide (Appendix A) was informed by the PPI group and the research team and piloted with one academic supervisor, two clinicians and one university Master's student. The designed topic guide shaped the direction of the conversation of the VLUs care in hospitals while allowing flexibility for participants to express their personal experiences.

Recruitment and data collection continued to the point of ‘information redundancy’ at which no new themes or codes were identified from the data from subsequent interviews [20]. To aid data analysis, field notes were collected and interviews were recorded digitally and transcribed verbatim. Transcripts were compared with the recordings to check accuracy and notes were made to identify initial impressions and reflections. Transcripts were downloaded as Microsoft Word documents and also uploaded to the data management software QSR N‐VIVIO Version 14.

### 2.4 Data Analysis

Interviews were analysed inductively using Thematic Analysis (TA) described by Braun and Clarke [21]. Braun and Clarke suggest this involves 6 stages: familiarising with the data, generating initial codes, searching for themes, reviewing themes, defining and naming themes [21]. To start with, the researcher (YL) familiarised herself with the data and noted down her initial thoughts; then processed inductively into generating initial codes, whereby words or short phrases from each transcript symbolically represented a summative, essence‐capturing subject or meaning within a sentence or paragraph [22, 23]. This process was accompanied by immersion in the content and attentive observation of emerging themes. This iterative engagement with the transcripts facilitated a more nuanced interpretation of participants' experiences and perspectives, thus enhancing the rigour of the analysis. In line with the phases of TA [21], multiple cycles of sorting, defining and refining codes and themes took place. During the mapping phase, the relationships and interactions between codes and themes were gradually established.

‘Participant recruitment and analysis were carried out simultaneously to enable constant comparison and identify similarities, differences and patterns within the data. During the data collection phase, as new data were coded and categorised into themes, these themes were compared against those previously developed. When subsequent interviews yielded no new themes and consistently reinforced existing patterns, the point of ‘data saturation’ was considered reached. Data saturation, also referred to as ‘information redundancy’, is a widely accepted concept in research studies employing thematic analysis [20]. At the same time, factors such as the conceptualisation of saturation were also taken into account [20]. This approach ensured a rich and comprehensive understanding of participants’ perspectives, thereby enhancing the trustworthiness and depth of the findings’.

### 2.5 Quality and Rigour

The trustworthiness of this study was rigorously evaluated. Credibility, transferability and confirmability of the data analysis were strengthened through several techniques [24]. These included obtaining a comprehensive sample from multiple hospitals across the United Kingdom, holding regular debriefing meetings with supervisors and conducting peer‐to‐peer scrutiny during the analysis phase. Interpretations of codes, subthemes and themes were challenged and verified by all supervisors (D.W., F.P., L.B., F.N.) and academic peers (F.F., C.S., J.M.). In addition, a Synthesised Member Checking (SMC) [25] was also used to validate, verify and assess the trustworthiness of the qualitative results [26].

All stages of data management were carefully documented in Word and NVivo software, including coding decisions and theme development. This provided a transparent record of analytic decision‐making and supported the dependability of findings. Attention was paid not only to dominant themes, but also to deviant cases that challenged emerging interpretations. Rather than treating these as anomalies, they were incorporated into the analysis as valuable insights into barriers and tensions in practice. This approach enhanced credibility by demonstrating that the analysis accounted for complexity and diversity within the data, rather than seeking neat consensus. Additionally, PPI members contributed to discussions about the codes and themes, assessing whether they resonated with their experiences. Preliminary key themes were presented at national and international conferences and shared with members of the study's Clinical and Academic Advisory panel.

### 2.6 Researcher Team and Reflexivity

The first author of this manuscript (Y.L., female, Tissue Viability Nurse Specialist [TVN]) was the primary researcher, conducting interviews and performing data analysis. Her clinical background provided an insider's perspective on the hospital environment, enabling her to relate her clinical experiences to staff participants' experiences, language and challenges. Y.L.'s relationships with interviewees were characterised by sharing professional language due to working in the same field of clinical practice. However, her clinical experience working with patients with VLUs could potentially present unconscious bias when conducting interviews and analysing data. To address this, the initial interview transcripts were reviewed by one academic supervisor (L.B.) to identify any leading questions. The supervisory meetings helped highlight areas for improvement and other opportunities for subsequent interviews. Y.L. kept a reflexive journal documenting reflections following each interview and her thought process when reading transcripts, generating initial codes and themes and identifying areas to refine her interviewing techniques.

### 2.7 Ethics Approval and Consent to Participate

Ethical approval was granted by the University of Leicester (UoL) Medical and Biological Sciences Research Ethics Committee (Project ID: 0257) and Health Research Authority (REC reference: 24/HRA/0574). Written informed consent was obtained before each interview.

## 3 Results

### 3.1 Participants Characteristics

Out of the 30 participants being interviewed, 19 participants' hospitals applied compression, of which 14 (74%) were female. Participants with over 20 years of work experience were the most common group (9, 48%), followed by those with 10–19 years of experience (7, 37%). Tissue Viability Nurses (Clinical Nurse Specialists providing expert advice in wound care) [27] were the most common practitioner type (8, 42%). They were followed by Vascular Nurses and Vascular Surgeons, each represented by three participants (16%). Vascular Nurses are Clinical Nurse Specialists who support Vascular services and care for patients with vascular conditions such as arterial and venous diseases, providing both inpatient and outpatient care [28]. Vascular surgeons are highly trained specialists who treat diseases of the vascular system [29]. The remaining participants included Senior Managers (2, 11%) and members of the coding and procurement team (3, 16%) who were involved in supporting the delivery of VLU care within their hospitals. All professions contributed valuable, though differing, perspectives, reflecting their unique roles in patient care. There were an equal number of participants from the North and Southeast regions of England (6, 32%), followed by the Midlands (2, 10.5%) and South (1, 5%), West (1, 5%) and Scotland respectively (1, 5%).

### 3.2 Main Findings

In the dataset of hospital staff where compression therapy is routinely applied, four key themes were generated:

1. Patients ‘Slip through the net’
2. Prioritisation within Hospital Care.
3. A ‘blind spot’ within Healthcare Systems.
4. Motivation to Deliver Care.

#### 3.2.1 Theme 1: Patients ‘Slip Through the Net’

Participants highlighted that inpatients with VLUs face unequal access to compression therapy both within and between hospitals. They gave various reasons for this, broadly categorised here under two sub‐themes: ‘Lottery of Care’ and ‘Lack of Ownership’.

##### 3.2.1.1 Lottery of Care

This theme referred to how access to compression therapy for inpatients with VLUs is often inconsistent from patients’ perspectives. Instead of a structured and standardised approach to treatment, inpatients with VLUs receive ‘Lottery of Care’, where access to care varies widely. The Lottery of Care was seen to operate at three levels: commissioning level, i.e., ‘post code lottery’, hospital decision‐making level, i.e., two‐tier system and the individual clinician level.

The ‘Lottery of Commissioning’ or ‘post code lottery’, refers to the concept that the quality or availability of the healthcare service can depend heavily on where someone lives (their postal code).

Nationally, the challenges are the variability, the post code lottery … you will certainly get a difference of care if you were a resident very local to my hub hospital compared to a resident in one of the spoke hospitals … because there is a lot of variability in … the ability to provide that care … (P07, Vascular Surgeon, Teaching Hospital).

A few participants explained that from commissioner's point of view, there are limited ‘monetary incentives’ to prioritise compression therapy within hospitals and that compression therapy does not generate immediate financial returns.

… A lot of these patients [patients with VLUs] are already at maximum comorbidities … [so] it's very rare that you get in any financial incentive by putting these patients into compression. There's no monetary incentive to get extra staff in place to manage these patients appropriately. (P06, Vascular Practitioner, Teaching Hospital)

Some hospitals employ vascular nurses primarily in outpatient settings rather than for inpatient care, leading to service fragmentation.

We do have vascular nurses, but they're sat in the Outpatient Department, so they don't come into the inpatients … they just happen to be in the same building, but they're funded differently [by the commissioners]. (P15, TVN, General Hospital).

The ‘Lottery of Hospital Decision‐Making’ refers to a two‐tier system where disparities exist in the provision of care, resources and access to services between different hospitals and wards under the same local management umbrella.

[There is] the main hospital [we provide compression therapy] … [But the] two other hospitals within our trust [that] … we would struggle to cover [compression therapy]. (P05, Vascular Practitioner, Teaching Hospital)

The ‘Lottery of Individual Clinician Decision‐Making’ refers to inconsistency in practices between hospital clinicians, even within the same professions. It emphasises how access to a fundamental treatment is not solely determined by clinical need but is significantly shaped by clinician knowledge, and their willingness to initiate and advocate for compression therapy:

It depends who the patient gets referred to … if they [patients] come to vascular … I [Vascular Nurse] will happily [apply compression]. But the vascular nurses from [another hospital] tend to say … the patients [should] be referred to Tissue Viability … [even] they know that the Tissue Viability Nurses can't do compression. (P22, Vascular Nurse Specialist, General Hospital)

In many cases, inpatients with VLUs receive unfair and inconsistent treatment due to the lottery of institutional capacity rather than patient needs.

At one point, they [Tissue Viability] did agree that they would put up to two patients into compression, no more than two because of their caseloads … If you're the third patient that comes in, you're not going to get that treatment [compression]. It's the inconsistency and it's unfair. (P06, Vascular Practitioner, Teaching Hospital)

Additionally, treatment decisions are also made based on hospitals' own priority rather than patient needs.

…Management is going to prioritise whatever they are getting pressure from above to deliver … We [Scottish Government] have given you Boards money. If your board chooses not to spend that money that way, that's their decision. (P18, Vascular Surgeon, Teaching Hospital)

##### 3.2.1.2 Lack of Ownership

Participants suggested that this disparity in care provided to inpatients with VLUs can be attributed to hospital clinicians' lacking clear ownership in managing these patients in hospitals. Several participants highlighted significant challenges in defining responsibility for VLU care, particularly when patients present with multiple comorbidities and are admitted under medical specialties. Consequently, wound care, including the application of compression therapy, may be neglected.

It doesn't fit in anybody's specific domain … When they're inpatients … with venous leg ulcers … under the care of the medical wards, because of their extensive comorbidities. You've got a patient who had a fall and got a fractured neck of femur and has been in compression. But nobody sees it as their responsibility. (P06, Vascular Practitioner, Teaching Hospital)

The absence of a designated team to assume responsibility for VLU management contributed to inconsistencies in care and missed opportunities for early intervention.

A further issue contributing to this lack of accountability is the ongoing conflict between vascular and tissue viability teams over which specialty should oversee VLU management in the hospitals.

The Vascular nurses told the ward [nurses] that all leg ulcers should be referred to Tissue Viability … and they changed the pathway … So, the Tissue Viability nurses should be assessing whether they are venous, also arterial… [However]. they [TVNs] think it's not their job … [so] these poor patients aren't getting the treatment they need … I don't know who will [take a more proactive approach]. I don't think anybody wants it. (P22, Vascular Nurse Specialist, General Hospital)

Many participants from the specialist teams indicated that another factor contributing to disparities in VLU management was the ward nurses' reluctance to take the ownership of managing patients with venous leg ulcers. They noted that ward nurses expressed fear regarding the application of compression bandages due to concerns about incorrect technique:

I think the fear … is … either the bandaging is done too tightly or not tightly enough to provide appropriate levels of compression … you know, ideal therapy … I think ultimately, it's just about the ability to safely apply compression … in accordance with how it should be applied. (P07, Vascular Surgeon, Teaching Hospital)

#### 3.2.2 Theme 2: Prioritisation Within Hospital Care

This theme refers to how certain diseases are given greater emphasis within healthcare systems. Participants identified arterial diseases being systematically prioritised over VLUs within the vascular surgical community. They revealed that arterial conditions are perceived as more urgent and critical, often commanding greater resources and attention, whereas VLUs are viewed as a lower priority:

Within the Vascular Surgical community, [venous] leg ulcers … are not considered to be the number one priority for the majority of vascular surgeons … I think internationally there is a hierarchical system … where arterial disease takes precedence … because a lot of the resource … is prioritised to patients with life and limb‐threatening conditions. (P07, Vascular Surgeon, Teaching Hospital)

Participants also highlighted a widespread national emphasis on reducing pressure ulcers in hospitals, driving hospital TVNs to prioritise pressure ulcer surveillance over VLUs management:

With the National drive reducing pressure ulcers … suddenly all the [hospital] Tissue Viability nurses have been counting … how many pressure ulcers do we have, and where did that come from? … I think the pressure ulcer agenda has been such a chain, ball and chain for us… we were lumbered with it. (P21, TVN, Teaching Hospital)

This prioritisation has led to a widespread perception among other healthcare professionals that TVNs are solely responsible for pressure ulcer management, rather than wound care more broadly.

The Chief Nurse thinks we only deal with pressure ulcers, [but] we've got other wounds as well … they're patients with leg ulcers … [But] we're only focused on [pressure ulcers]. If the chief nurse gets an incident report escalated to you, you [TVNs] stop everything. (P10, TVN, General Hospital)

Another key disparity identified in this study is the concerns over resource allocation between venous ulcer care and pressure ulcer care. A few participants reported that patients with longstanding VLUs do not receive the same level of urgency or intervention as patients with pressure ulcers.

If somebody [patient] came in and didn't have the correct mattress or didn't have the correct equipment at home and came in with a pressure ulcer, everybody would be jumping all over it. Yeah, we've had somebody come in with leg ulcers for 10 years, no one's done the right thing despite NICE guidelines … the National Wound Care Strategy Programme and NHS Long Term Plan. All of the things that are there to say this should be happening and we just continue [not apply compression therapy for these patients]. (P15, TVN, General Hospital)

#### 3.2.3 Theme 3:A ‘Blind Spot’ Within Healthcare Systems

Participants identified that failing to implement compression therapy for inpatients with VLUs has created a critical underlying ‘blind spot’ within the relevant NHS healthcare systems. They described the detrimental impact of not initiating compression therapy on wound healing:

The wound would deteriorate, and they'll be setbacks significantly regarding their healing trajectory … You're going to delay the wound healing … [by] not treating that underlying venous aetiology … (P06, Vascular Practitioner, Teaching Hospital)

Participants also highlighted a critical oversight within the NHS, where the failure to prioritise evidence‐based compression therapy leads to not only prolonged patient suffering but also increased healthcare costs. This systemic neglect reflects a ‘blind spot’ in healthcare planning and resource allocation, ultimately exacerbating the financial and clinical burden associated with VLUs:

It's more expensive to not control these ulcers with compression. Because it will be wet. The non‐compressive bandage will not do any good to them. It might prolong their period of nonhealing … I think there's a blind spot in our NHS care. (P14, Vascular Surgeon, Teaching Hospital)

Some participants stated venous ulcer care remain largely ‘invisible’ within acute hospital settings.

Every time something came out from the National Wound Care Strategy … in relation to leg ulceration, its primary focus was community. Yet all of those patients come through acute sector [at some point] … So … the cost of the burden is actually massively more than what's been suggested, because we're invisible! Wound care in acute services is invisible! (P08, TVN, Teaching Hospital)

The failure to adopt a holistic, long‐term financial perspective in managing VLUs was also echoed by a senior manager.

… within healthcare … they don't look at the bigger picture … if you give the right therapy then, that will prevent the patient coming back in [to hospitals]. [Otherwise] … deterioration will occur … actually that costs more in the long term. And yes, it may not be that particular hospital that bears the cost, but overall, it's still part of the same public purse, isn't it? (P19, Senior Manager, Teaching Hospital)

Some participants further exposed another significant barrier to improving VLU care within the NHS as the absence of robust and accurate VLU data. The lack of comprehensive datasets for lower limb conditions hinders their ability to assess prevalence, monitor treatment outcomes and allocate resources effectively.

We don't have really robust data … So the data that I do have on that is the data that I get from my referrals … this is the problem … if you don't have a dataset for your particular area for lower limb … and you say the prevalence, it makes it really difficult to be able to send those figures.’ (P15, TVN, General Hospital)

The lack of comprehensive data limits the ability to assess the true prevalence of VLUs, monitor treatment outcomes and allocate appropriate resources. This lack of visibility contributes to the under‐prioritisation of VLU care, as decision‐makers often rely on data‐driven evidence to justify healthcare investments. Without reliable statistics, VLUs may not be recognised as a critical issue requiring targeted intervention. A contributing factor to the invisibility of VLU data is the complexity of medical coding systems.

When they code, it can be quite tricky to code, because there isn't actually like leg ulcer [coding] as such … (P15, TVN, General Hospital)

#### 3.2.4 Theme 4: Motivation to Deliver Care

Despite systemic and structural challenges, many participants exhibited a deep commitment to providing compression therapy, driven by their inherent dedication and ethical obligation towards patient quality of care. This is demonstrated through their passion, motivation and moral responsibility alongside both top‐down and bottom‐up support from their senior management and stakeholders.

##### 3.2.4.1 Passion, Motivation and Moral Responsibility

Participants demonstrated profound passion, motivation and moral responsibility, which appeared to play a crucial role in shaping clinicians' engagement in VLU care in their hospitals, even in the absence of formal recognition or structured support:

Despite us not having any funding, we decided to go anyway. That's down to the team, that's down to the TVNs…… actually the easy option would be to say we've got no funding, that's more work, we're not going to do it, and it's going to cause a lot of ripples. But we didn't, we decided as a team it was the right thing to do (apply compression). (P15, TVN, General Hospital)

Other clinicians expressed the passion by recognising the importance of assessing patients in a timely manner.

If they [patients with VLUs] are in hospital, why do they not just get assessed in hospital and then it's done and then we'll know what the plan is. That would be the easiest thing [for patients]. (P22, Vascular Nurse Specialist, General Hospital)

Several clinicians highlighted the need for cultural change in hospital settings regarding VLU management.

… This [applying compression therapy] is a cultural change … The acute have never seen it as something that they manage… it seems to be that the acutes have never really done compression therapy. So that has to change. (P15, TVN, General Hospital)

Their strong desire to address the widespread issue for inpatients with VLUs could be due to the fact that they recognise patients' need and service needs.

… We see a lot of patients with venous leg ulcers … high volume of at least 20% of their patients on the ward may have them [venous leg ulcers] at one time … (P19, Senior Manager, Teaching hospital)

Additionally, some clinicians believe they should ‘take responsibility for not acting, not to do *something*’ *because they are* ‘*not competent*’. *They* challenged the notion of avoiding compression therapy due to lack of training or uncertainty. Other clinicians drew a direct parallel between compression therapy and other fundamental treatments: ‘*We are* … *looking at doing compression bandaging as a treatment* … *you can't just ignore it* … *that's there to treat the ulcer. You know, if you've got a chest infection, you would take antibiotics. If you've got a leg ulcer, you have compression*.’ (P23, TVN, Teaching Hospital). This comparison again featured the necessity of compression therapy as a standard of care rather than an optional intervention.

Ultimately, clinicians derive a sense of pride in their team and place their trust in them due to their unwavering passion and moral responsibility. They possess a strong sense of pride, teamwork, intrinsic motivation and joy in providing compression therapy to patients with VLUs in their hospitals:

I'm very proud of my team. I have to say because they have absolutely taken it [applying compression therapy] on … because it's in the patients' best interest … That's what gives us most of us joy at work, isn't it? (P08, TVN, Teaching Hospital)

##### 3.2.4.2 Top‐Down Support

Several participants attributed the successful implementation of compression therapy to sustained support from senior leaders within their respective organisations. This support enabled the TVN teams to overcome organisational barriers and facilitated engagement with other healthcare professionals.

I have been hugely supported by my Head of Service … she understands leg ulcers … knows what a difference compression can make. So … having her as a stakeholder is … pivotal … because she has a lot of buy‐in in senior management and with the trust. (P15, TVN, General Hospital)

The importance of institutional culture in promoting wound care was also highlighted. One senior manager reflected on the broader organisational culture that recognised wound care as a priority.

As a whole within the trust, I think wound care is seen as important … you absolutely need that, because you know patients aren't getting the right care. (P19, Senior Manager, Teaching Hospital)

Some participants described engaging key organisational stakeholders to ensure that VLU management was approached from multiple perspectives.

… We had a lot of stakeholders involved in this … We had a stakeholder engagement meeting … [like] a listening of engagement event … on how we can improve things. (P08, TVN, Teaching Hospital)

Other TVNs highlighted advantages in reporting directly to senior management, which provided them with the necessary institutional support to implement compression therapy, ‘*I [TVN] had great support because I report quite high up in the nursing structure. And so therefore I had support*.’ (P21, TVN, Teaching Hospital).

To maintain alignment and ensure ongoing stakeholder engagement, participants organised regular update meetings to discuss progress and address challenges: ‘*Every month, there's a big meeting … to update everybody [all stakeholders] once a month*.’ (P23, TVN, Teaching Hospital). These regular meetings fostered a culture of continuous improvement and accountability, where feedback was actively sought and addressed.

On the other hand, some participants also recognised the negative aspect of the Top‐down support where, while political initiatives, such as the All‐Party Parliamentary Group, exist to address healthcare inequalities, they often lack meaningful impact:

I think … the All‐Party Parliamentary Group are quite helpful. I say quite helpful, not very helpful because … some [politicians] are just going there for the photo opportunities and then they leave after three minutes. (P14, Vascular Surgeon, Teaching Hospital).

##### 3.2.4.3 Bottom‐Up Support

Participant identified a shared vision and collective commitment among team members as key factors in promoting the adoption of compression therapy:

There's never going to be a perfect starting point … If we waited for everything in the end, I think we'd still be waiting … The team have been so supportive, and we've got a shared vision of promoting patients with lower limb wounds …we (as a team) are going to sort of learn a bit on the way, because some of those things we would have never known if we didn't try. (P15, TVN, General Hospital)

Other participants recognised the importance of empowering ward nurses by equipping them with the necessary skills and confidence to apply compression therapy independently:

… they [ward nurses] will learn how to do it and they will do it properly … [The ward] need to have enough nurses that know how to do compression bandages … we tissue viability do all of the assessment …, [ward nurses] need to follow it up [and reapply the compression bandages] …. (P21, TVN, Teaching Hospital)

Several TVNs actively promoted the cost‐effectiveness of compression therapy as a persuasive argument to gain wider organisational buy‐in:

…It's quite interesting that there is actually more cost effective to use compression twice a week … reduced compression actually is more cost effective by the looks of our data at the moment than not [applying compression therapy] … it's selling the benefits across the whole organisation … (P08, TVN, Teaching Hospital)

Some TVNs recognised the need to identify training needs, take away the fear about compression therapy:

The biggest change that we've done is to change the way we do the education and make this skill for acute nurses only … we're trying to make things as easy as possible … [and] simplified everything down … we've got one compression [bandage] … one practical session … one online session … it takes away that fear [about compression] and allows them [ward nurses] to know exactly what they're doing … (P23, TVN, Teaching hospital)

Senior managers also emphasised the importance of using incident reporting systems to promote accountability and ensure timely intervention when standards of care were not met.

Safety is a big driver in this trust … if patients aren't getting the care they need … we do use incident reporting [system] … incidents are about learning … (P19, Senior Manager, Teaching Hospital)

## 4 Discussion

This study explored clinicians, senior managers and other hospital staff's perceptions regarding the application of compression therapy in acute hospitals across the UK where compression therapy could be accessed by inpatients. These results indicate a systematic failure to apply compression therapy in acute hospitals. Patient access to compression therapy is influenced by reported variabilities in healthcare service provision such as funding structures, resource allocation, commissioning decisions across hospital settings, workforce capacity and individual clinician decision‐making. In addition, the disparity in prioritisation between pressure ulcers and VLUs raises broader concerns about healthcare equity. As a result, clinicians across multiple professional groups perceive this as a ‘blind spot’ in the healthcare system, which could lead to delayed wound healing, increased long‐term healthcare costs and exacerbated financial and clinical burden associated with venous ulcer care. Nevertheless, clinicians describe their inherent motivation and sense of duty as pushing them to deliver best‐practice care despite systemic challenges.

Inpatients with VLUs are seen to ‘slip through the net’ due to a ‘Lottery of Care’. The ‘Lottery of Care’ suggests access to compression therapy in hospitals is inconsistent, with patients receiving treatment depending on healthcare service commissioning, resource allocation and clinicians’ knowledge. Hospitals with greater financial and workforce capacity can prioritise specialist‐led care, while those with fewer resources struggle to provide comprehensive treatment options for patients with VLUs [30].

The lottery of commissioning links with previous research [3] that regional disparities in healthcare funding and commissioning contribute to unequal access to specialist services, including wound care treatment for VLUs. This variability in practice directly contributes to inequities in care, echoing broader systemic disparities within the NHS and highlighting the complexities of translating evidence into consistent clinical practice. In addition, the management of VLUs in inpatient settings is hindered by a lack of clear ownership and professional disputes. The lack of accountability in inpatient care is particularly concerning, as evidence suggests that delayed or inconsistent application of compression therapy leads to prolonged healing times and an increased risk of complications [31, 32, 33]. The perceived fragmentation of care responsibilities among different professional groups reinforces the need for clearly defined care pathways and designated teams to oversee VLU management.

Vascular surgery prioritises arterial diseases over VLUs despite their prevalence and economic burden, resulting in inconsistent treatment and poorer patient outcomes. Hospital settings were also seen to prioritise pressure ulcer prevention and management over VLUs care. National policies and quality improvement initiatives identified pressure ulcer reduction as a key patient safety indicator [34]. This prioritisation is seen to be reinforced by institutional pressures and reporting structures due to national quality targets, such as Commissioning for Quality and Innovation (CQUIN) schemes [35]. As a result, hospital TVNs' workloads were seen to shift to increased pressure ulcer surveillance and reporting, often at the expense of managing other chronic wounds like VLUs [35]. This prioritisation is seen to be reinforced by care for Quality and Innovation (CQUIN) schemes. Consequently, VLUs, a significant burden on patients and healthcare systems, are seen to receive inadequate attention in hospitals.

The ‘Lottery of Care’, ‘Lack of Ownership’ and the de‐prioritisation of VLUs care in hospitals lead clinicians to recognise it as a ‘blind spot’ in healthcare. The ‘blind spot’ is reflected in patient consequences, then made invisible such as delayed ulcer healing, increased morbidity and significant patient distress [31, 32, 33]. The lack of effective compression therapy in acute hospitals is not only clinically detrimental but also financially inefficient. The NHS faces a substantial economic burden due to prolonged wound chronicity, frequent hospital readmissions and increased nursing time for wound management [3]. Another major barrier to improving VLU management is the lack of robust data on VLUs prevalence, treatment outcomes and resource utilisation. Participants express frustration over the difficulty in obtaining accurate epidemiological data due to inconsistencies in medical coding, which result in underreporting of VLUs within hospital records. Similar challenges have been identified in that inadequate coding practices have led to gaps in healthcare, making it difficult to track patient outcomes and assess service effectiveness [36]. Without reliable figures from hospital settings, VLUs are not perceived as a priority area for resource allocation, leading to continued underfunding and inadequate service provision. Addressing this issue requires a systematic approach to data collection, including the implementation of standardised coding practices and integrated electronic health records [36]. Strengthening the visibility of VLUs within healthcare data is crucial for driving policy changes and ensuring that compression therapy is embedded as a routine aspect of inpatient care.

Findings also showed the significant role of passion, motivation and moral responsibility in supporting clinicians to implement compression therapy for inpatients with VLUs despite systemic barriers. Several participants emphasised that leadership in VLU management may depend less on professional background than on passion and commitment to improving patient outcomes. Research on healthcare leadership also suggests that fostering ‘champions’ within multidisciplinary teams is key to implementing best practices and driving quality improvement [37].

Additionally, engaging a broad range of stakeholders, including senior managers, ward nurses, vascular specialists and other relevant departments, ensured that diverse perspectives were considered and addressed during the implementation process. This approach is consistent with best practices in change management, which emphasise the importance of stakeholder buy‐in and shared ownership in driving successful interventions [38].

### 4.1 Implications of the Findings

The findings suggest the following implications: firstly, hospital staff see standardising care pathways and protocols for VLU management within inpatient settings as crucial for the application of compression therapy in hospitals [39]. Secondly, staff see ensuring adequate resourcing of specialist teams as essential to meet the needs of the patient population and avoid the necessity for rationing care based on capacity rather than clinical need. Finally, addressing the lack of VLU management education requires structured educational programs, mentorship opportunities and clear guidelines. Investing in comprehensive education and training programs for all relevant healthcare professionals can enhance their knowledge, skills and confidence in delivering compression therapy, leading to more equitable and evidence‐based care for inpatients with VLUs. In addition, ensuring more widespread application of compression therapy in acute hospitals requires a systemic culture shift in VLUs care within acute care settings, alongside structured interventions to educate both healthcare providers and patients on the necessity of timely compression therapy. Future research will explore how the VLUs care is delivered for inpatients in hospital settings. This will help design targeted strategies to integrate compression therapy into routine hospital care and strengthen patient advocacy in wound management.

### 4.2 Strengths and Limitations

The strength of this study lies in its comprehensive exploration of the reported experiences of a diverse range of clinicians, senior managers and other hospital staff who are involved in the application of compression therapy for inpatients with VLUs. However, the limitation of the study is that it solely represents the opinions of the participants and does not necessarily reflect their actions. Additionally, the management of VLUs requires clinicians from both the hospital and community teams and the experiences of all parties are necessary to fully comprehend the impact of applying compression therapy for patients with VLUs. Furthermore, the recruitment for this study was primarily through self‐referral, which presents a potential bias as participants who volunteered to participate are more likely to have a specific interest in venous leg ulcers. The professional networks sought may be more likely to reach particular types of clinicians who have a greater interest and involvement in this area. This study presents participants' perspectives and experiences, potentially influenced by social desirability bias, which may limit the representation of the full picture. It may also provide a different perspective than other approaches, such as ethnographic studies, which can offer a more comprehensive and contextualised portrayal of hospitals' dynamics and practices. Another limitation is that patient experiences may differ from those perceived by clinicians.

## 5 Conclusion

The study identified systemic inequities, professional roles and responsibilities, organisational priorities and passion, motivation and moral responsibility as pivotal factors influencing the application of compression therapy for inpatients with VLUs. While certain factors presented challenges contributing to treatment delays and variability in care, ultimately impacting patient outcomes, others such as passion, motivation and moral responsibility presented opportunities for compression therapy application within hospitals. The study highlighted the urgent need to address the systemic challenges inherent in inpatient VLU care. Establishing a standardised, evidence‐based care pathway with clearly defined roles and responsibilities is paramount to ensure that all inpatients with VLUs receive timely and appropriate VLU management. Implementing structured training programs, promoting interdisciplinary collaboration and establishing comprehensive guidelines for VLU care can significantly enhance patient outcomes and mitigate variability in treatment approaches.

## Funding

Yaping Lian was funded by a National Institute for Health and Care Research (NIHR) Doctoral Clinical and Practitioner Academic Fellowship (DCAF) (NIHR302890). The views expressed are those of the authors and not necessarily those of the NIHR or the Department of Health and Social Care.

## Ethics Statement

Ethical approval was granted by the University of Leicester (UoL) Medical and Biological Sciences Research Ethics Committee (Project ID: 0257) and Health Research Authority (REC reference: 24/HRA/0574).

## Consent

Written consent was gained before each interview.

## Conflicts of Interest

The authors declare no conflicts of interest.

## Supporting information


**Appendix A:** IWJ 70810‐Sup‐0001‐ Supinfo

## Data Availability

The data that support the findings of this study are available on request from the corresponding author. The data are not publicly available due to privacy or ethical restrictions.
