# Supplementary material for: A Qualitative Study Examining the Application of Compression Therapy for Inpatients With Venous Leg Ulcers—Perspectives of Hospital Staff Where It Is Routinely Applied
Source: Int Wound J. 2026 Feb 3;23(2):e70810. doi: 10.1111/iwj.70810 (PMC12868380; doi:10.1111/iwj.70810)
Supplement: Supplementary file 1 — Appendix A: IWJ 70810‐Sup‐0001‐ Supinfo [file IWJ-23-e70810-s001.docx]

## OCTOPUS study

**Optimising Compression Therapy fOr inPatients with venous UlcerS**

**Interview Topic Guide**

**Welcome and introduction**

Thank you for agreeing to take part in the interview. I appreciate you are very busy, so the interview will not take longer than 1 hour.

I would like to highlight the **confidentiality** of the interview that everything you tell me will be kept confidential unless you tell me something that indicates the danger to yourself or others or poor practises, in which case, it would need to be reported, but I will discuss that with you.

- The recording will be deleted after being transcribed.
- You won’t be identified individually in any report.
- All information will be anonymised.
- **We will not tell anyone else including your employer organisation, what you tell us as an individual.**
- All your views are of value to us. There are no right or wrong answers.
- Please ask me to clarify if the questions isn’t clear.
- We remind you not to share any personal or patient identifying information during this interview.
- Occasionally, I might look down to take notes, please assured that I am still actively listening.

| What are your experiences of managing leg ulcers in your hospital? |  |
| --- | --- |
| What treatment did you use in your hospital? |  |
| What kind of policy and guidelines do you have for the care of patients with leg ulcers in your hospital? |  |
| How did you learn about the skills and knowledge for patients with venous leg ulcers? |  |
| What are your experiences of using compression therapy in hospitals?  Some experience that it can be difficult to implement compression therapy in hospitals.  What do you think might help address these views? |  |
| How confident do you feel about the use of compression in your hospital? |  |
| What other areas do you think might have an impact on the use of compression therapy in your hospital? |  |

**Anything not covered?** Is there anything that we haven’t covered in the interview that you think we should know or think about?

**Closing and thanks –** I know a lot of what we talk about are kind of sensitive, so just would like to check that you are ok and that you are still happy for me to use all the information provided. If you like, you could ask to erase any sections of the recording.

Thank them for their time and contribution.
